# Salt‐Inducible Kinase 2‐Triggered Release of Its Inhibitor from Hydrogel to Suppress Ovarian Cancer Metastasis

**DOI:** 10.1002/advs.202202260

**Published:** 2022-05-26

**Authors:** Yue Hua, Han Yin, Xiaoyang Liu, Jinbing Xie, Wenjun Zhan, Gaolin Liang, Yang Shen

**Affiliations:** ^1^ Department of Obstetrics and Gynaecology Zhongda Hospital School of Medicine Southeast University Nanjing Jiangsu 210009 China; ^2^ State Key Laboratory of Bioelectronics School of Biological Science and Medical Engineering Southeast University 2 Sipailou Road Nanjing 210096 China

**Keywords:** antimetastasis, hydrogels, ovarian cancer, responsive release, salt‐inducible kinase 2

## Abstract

Salt‐inducible kinase 2 (SIK2) is a promising target for ovarian cancer therapy due to its critical role in tumorigenesis and progression. Currently available SIK2 inhibitors have shown remarkable therapeutic effects on ovarian cancers in preclinical studies. However, direct administration of the SIK2 inhibitors may bring significant off‐target effect, limiting their clinical applications. In this work, by rational design of a hydrogelator Nap‐Phe‐Phe‐Glu‐Glu‐Leu‐Tyr‐Arg‐Thr‐Gln‐Ser‐Ser‐Ser‐Asn‐Leu‐OH (**Nap‐S**) to coassemble a SIK2 inhibitor HG‐9‐91‐01 (**HG**), a SIK2‐responsive supramolecular hydrogel (**Gel Nap‐S+HG**) for local administration and SIK2‐responsive release of **HG** is reported to efficiently suppress ovarian cancer metastasis. Under the activation of SIK2 overexpressed in ovarian cancers, **Nap‐S** in the hydrogel is phosphorylated to yield hydrophilic Nap‐Phe‐Phe‐Glu‐Glu‐Leu‐Tyr‐Arg‐Thr‐Gln‐Ser(H_2_PO_3_)‐Ser‐Ser‐Asn‐Leu (**Nap‐Sp**), triggering the disassembly of the hydrogel and a responsive release of the inhibitor. Cell experiments indicate that sustained release of **HG** from **Gel Nap‐S+HG** induce a prominent therapeutic effect on cancer cells by inhibiting SIK2 and phosphorylation of their downstream signaling molecules. Animal experiments demonstrate that, compared with those tumor model mice treated with free **HG**, **Gel Nap‐S+HG**‐treatment mice show an enhanced inhibition on ovarian tumor growth and metastasis. It is anticipated that the **Gel Nap‐S+HG** can be applied for ovarian cancer therapy in clinic in the near future.

## Graphical abstract

Upon activation by salt‐inducible kinase 2 (SIK2) overexpressed in ovarian cancers, hydrogelators in the HG‐9‐91‐01‐coassembled nanofibers will be efficiently converted to their hydrophilic phosphates, triggering the disassembly of the nanofibers and the sustained release of HG‐9‐91‐01. The released HG‐9‐91‐01 will in turn down‐regulate SIK2 activity and consequently inhibit its downstream proteins to efficiently promote apoptosis of ovarian cancer cells.

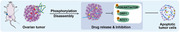

## 1 Introduction

Ovarian cancer is the third most common but the deadliest gynecologic cancer.^[^ ^1^ ^]^ High degree of malignancy, rapid development, and difficulty of detection at early stage have become prominent features of ovarian cancer.^[^ ^2^ ^]^ Consequently, the majority of ovarian cancer cases are diagnosed at advanced stages.^[^ ^3^ ^]^ For ovarian cancer patients, the preferred treatment option is to perform primary debulking surgery to achieve R0 degree, followed by chemotherapy.^[^ ^4^ ^]^ While for those cannot achieve R0 resection, neoadjuvant chemotherapy is needed to treat the cancers to get their diseases completely or partially responsive. Paclitaxel combined with carboplatin remains the first‐line chemotherapy for ovarian cancer.^[^ ^5^ ^]^ Nevertheless, the recurrence rate of ovarian cancer after first‐line therapy is as high as 75%.^[^ ^6^ ^]^ Moreover, these recurrent ovarian cancers are prone to paclitaxel/platinum resistance and subject to a series of serious complications such as anemia, neurotoxicity, and reduced life quality, leading to a 5‐year survival rate less than 50%.^[^ ^7^ ^]^ Recently, the advent of targeted drugs (e.g., bevacizumab,^[^ ^8^ ^]^ PARP inhibitor,^[^ ^9^ ^]^ and PD‐1/L1 inhibitor^[^ ^10^ ^]^) provides alternative options for ovarian cancer therapy. However, these treatments only benefit a small part of ovarian cancer patients, especially for those with breast cancer (BRCA) mutations.^[^ ^11^ ^]^ In addition, these expensive drugs have certain hematologic toxicities to the patients such as anemia, neutropenia, and thrombocytopenia.^[^ ^12^ ^]^ With the development of cell and molecular biology, increasing numbers of key signaling molecules (e.g., enzymes, growth factor receptors, and signal transducers and activators) were evidenced to be directly involved in tumorigenesis.^[^ ^13^ ^]^ Thus, developing new therapies that target these signaling molecules might be a promising strategy for ovarian cancer treatment.^[^ ^14^ ^]^

Recently, salt‐inducible kinase 2 (SIK2), a serine/threonine protein kinase which belongs to adenosine monophosphate‐activated protein kinase (AMPK) subfamily, is considered as an attractive and potential therapeutic target.^[^ ^15^ ^]^ Accumulating studies indicated that SIK2 is overexpressed in both primary ovarian cancer tissues and metastatic foci, and plays a crucial role in tumor occurrence and progression.^[^ ^16^ ^]^ On one hand, SIK2 up‐regulates glucose level through p85*α* phosphorylation‐activated PI3K/AKT/mTOR pathway and dynamin related protein 1 (Drp1) phosphorylation‐mediated mitochondrial fission, which further induces tumorigenesis and promotes tumor metastasis.^[^ ^17^ ^]^ On the other hand, SIK2 enhances intracellular fatty acid oxidation through acetyl‐CoA carboxylase 1 (ACC1) phosphorylation, thereby promoting adipocyte‐mediated proliferation and metastasis of ovarian cancer cells.^[^ ^16^ ^]^ Thus, increasing efforts have been devoted to developing SIK2 inhibitors to prevent ovarian cancer growth and metastasis. The SIK inhibitors in current use, such as ARN‐3236, YKL‐05‐099, YKL 06‐061, and HG‐9‐91‐01 (**HG**), have shown great effects on SIK2 activity inhibition.^[^ ^18^ ^]^ Among them, **HG** shows the best selectivity to SIKs among the members of the AMPK‐related kinase subfamily.^[^ ^19^ ^]^ In vitro assays indicated that **HG** has succeeded in suppressing ovarian cancer metastasis by targeting the adenosine triphosphate (ATP)‐binding site as well as a small hydrophobic pocket near this site.^[^ ^19a^ ^]^ Nevertheless, in those in vivo cases, the therapeutic efficacy of this small molecule SIK2 inhibitor is greatly compromised due to its low bioavailability, short serum half‐life, and nonignorable cellular toxicity, making it unsuitable for direct administration.^[^ ^20^ ^]^ Naturally we think that a “smart” strategy of local administration and sustained release of these SIK2 inhibitors should be an ideal strategy to enhance their therapeutic efficacy as well as reduce their systematic toxicity at a minimal dosage.

The newly emerging supramolecular hydrogels are considered as the ideal drug delivery systems to achieve above goal.^[^ ^21^ ^]^ Supramolecular hydrogels are formed by noncovalent self‐assembly of the hydrogelators (e.g., small organic molecules, saccharides, nucleobase derivatives, and peptides).^[^ ^22^ ^]^ Among which, peptide‐based hydrogel is of unique advantage due to the inherent intrinsic properties of the peptide hydrogelator (e.g., simple synthesis and easy functionalization) and the hydrogel (e.g., good biocompatibility and tunable biodegradability).^[^ ^23^ ^]^ In addition, drugs can be efficiently and stably loaded into such hydrogel by coassembly with or covalent attaching to the hydrogelators. These features make the peptide hydrogel an ideal platform for local administration of drugs to minimize their off‐target‐related adverse effect, while in the meantime increase their bioavailability.^[^ ^24^ ^]^ Importantly, by utilizing valuable endogenous stimuli to activate the hydrogel, smart and sustained release of encapsulated drugs can be achieved.^[^ ^25^ ^]^ Over the last few years, kinases overexpressed in disease sites have been used as powerful stimuli to induce gel‐to‐solution transition of supramolecular hydrogels for controlled drug delivery.^[^ ^26^ ^]^ For example, we designed “smart” tyrosine kinase‐responsive peptide‐based hydrogels for local administration and sustained release of encapsulated drugs to overcome organ transplantation rejection^[^ ^27^ ^]^ or delay ovarian aging.^[^ ^28^ ^]^

**Scheme 1:**
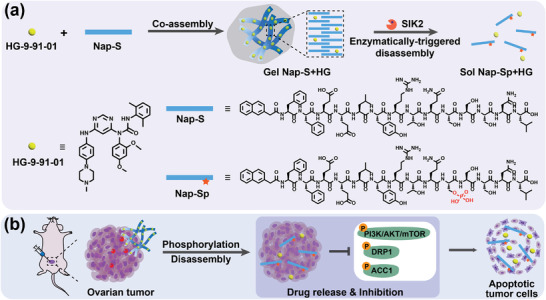
Conceptual illustrations and the chemicals in this work. a) Schematic illustration of the formation of **Gel Nap‐S+HG,** and its disassembly to release **HG** by SIK2‐triggered phosphorylation of **Nap‐S** to **Nap‐Sp**. b) Local delivery of **Gel Nap‐S+HG** for SIK2‐responsive and sustained release of **HG** to efficiently promote apoptosis of ovarian cancer cells by inhibiting the phosphorylation of SIK2 downstream proteins.

Herein, we intended to develop a “smart” kinase‐triggered gel‐to‐solution transition strategy for local delivery and controlled release of SIK2 inhibitors at ovarian tumor sites to efficiently inhibit proliferation and metastasis of the tumor. Specifically, we rationally designed a hydrogelator Nap‐Phe‐Phe‐Glu‐Glu‐Leu‐Arg‐Thr‐Gln‐Ser‐Ser‐Ser‐Asn‐Leu‐OH (**Nap‐S**), which consists of i) a commonly used self‐assembling motif Nap‐Phe‐Phe, ii) a dipeptide Glu‐Glu spacer with negative charges to regulate overall charge balance of the gelator, and iii) a specific peptide substrate Leu‐Arg‐Thr‐Gln‐Ser‐Ser‐Ser‐Asn‐Leu‐OH (LYRTQSSSNL) for SIK2‐recognized phosphorylation. We chose LYRTQSSSNL because it is the exact SIK2 phosphorylation site at the downstream protein p85*α* of SIK2.^[^ ^16^ ^]^ Supramolecular hydrogel **Nap‐S** (**Gel Nap‐S**) could be easily obtained via a heating‐cooling method. Since the SIK2 inhibitor **HG** contains four aromatic groups, it readily coassembles with **Nap‐S** to yield the hydrogel **Nap‐S**+**HG** (**Gel Nap‐S+HG**). We propose that, upon SIK2 activation, **Nap‐S** in the hydrogels will be efficiently converted to its hydrophilic phosphate Nap‐Phe‐Phe‐Glu‐Glu‐Leu‐Tyr‐Arg‐Thr‐Gln‐Ser(H_2_PO_3_)‐Ser‐Ser‐Asn‐Leu (**Nap‐Sp**), triggering the disassembly of the hydrogels and the release of **HG** in a sustainable manner (**Scheme** **1**a). After the hydrogel is locally injected, SIK2‐reponsive release of **HG** at the ovarian tumor site is achieved (Scheme 1b). The released **HG** will in turn down‐regulate SIK2 activity and consequently inhibit its downstream proteins (e.g., mTOR, DRP1, and ACC1) to efficiently suppress ovarian cancer proliferation and metastasis (Scheme 1b).

## 2 Results and Discussion

### 2.1 Preparation and Characterizations of the Hydrogels

First, we use solid phase peptide synthesis (SPPS) to produce the designed hydrogelator **Nap‐S** and its phosphorylation product **Nap‐Sp** (Scheme 1, Supporting Information). After purifications with high‐performance liquid chromatography (HPLC), the structures of the peptides were confirmed with matrix‐assisted laser desorption ionization time‐of‐flight (MALDI‐TOF) mass spectra and nuclear magnetic resonance (NMR) spectra (^1^H NMR and ^13^C NMR) (Figures S1–S4, Supporting Information). Afterward, we tested their gelation property upon heating–cooling process. Specifically, 1.0 wt% **Nap‐S** or **Nap‐Sp** in phosphate‐buffered saline (PBS, 0.01 m, pH 7.4) solution was heated up to 65 °C, and then cooled down to room temperature. As shown in Figure S5a (Supporting Information), **Nap‐S** solution formed a stable and transparent supramolecular hydrogel (**Gel Nap‐S**), illustrating good self‐assembling property of compound **Nap‐S**. However, upon heating and cooling, solution **Nap‐Sp** remained a solution (Figure S5b, Supporting Information). These results suggest that **Gel Nap‐S** may undergo a gel–sol transition after the gelator **Nap‐S** is phosphorylated to **Nap‐Sp** by SIK2. Moreover, we evaluated the coassembly capacity of **HG** by mixing it with **Nap‐S** at different **HG**/**Nap‐S** molar ratios (ranging from 1:500 to 1:7.8, Table S1, Supporting Information), followed by heating–cooling process as above. As expected, similar transparent hydrogels were obtained, suggesting that **HG** coassembled well with **Nap‐S** and thus was stably encapsulated in the hydrogel (Figure S6, Supporting Information). Based on the results of preliminary cytotoxicity experiments (Figure S7, Supporting Information), we found that hydrogel with a **HG**/**Nap‐S** ratio of 1:125 showed excellent anticancer activity. However, coassembled hydrogels with higher **HG**/**Nap‐S** ratios were too cytotoxic to SKOv3‐SIK2 cells and not suitable for further experiments. Thus, an optimized coassembled hydrogel **Gel Nap‐S+HG** with a molar ratio 1:125 of **HG**:**Nap‐S** was chosen for further experiments.

**Figure 1:**
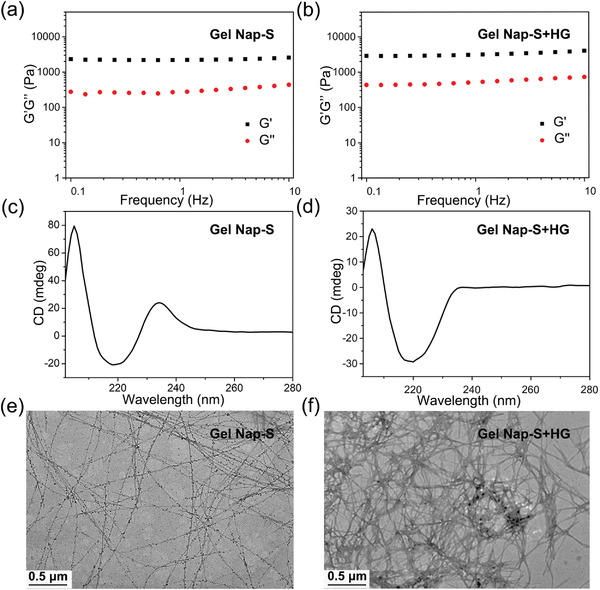
Characterizations of the hydrogels. a,b) Frequency dependence of the dynamic storage moduli (*G*´) and the loss moduli (*G*´´) of **Gel Nap‐S** and **Gel Nap‐S+HG** (molar ratio: **Nap‐S**/**HG** = 125/1) at 1.0 wt% in PBS (25 °C, strain: 1.0%). c,d) CD spectra of **Gel Nap‐S** and **Gel Nap‐S+HG** (molar ratio: **Nap‐S**/**HG** = 125/1) at 1.0 wt% in PB (pH 7.4). e,f) TEM images of 1.0 wt% **Gel Nap‐S** and **Gel Nap‐S+HG** (molar ratio: **Nap‐S**/**HG** = 125/1), respectively.

Next, physical properties of **Gel Nap‐S** and **Gel Nap‐S+HG** were evaluated using a series of experiments. First, dynamic rheological measurements were performed to study the viscoelastic properties of the obtained hydrogels. As illustrated in **Figure** **1**a,b; and Figure S8 in the Supporting Information, the storage modulus (*G*´) of **Gel Nap‐S** and **Gel Nap‐S+HG** were significantly higher than their loss modulus (*G*´´) in the investigated frequency range (0.1–10% Hz) and strain range (0.1–10%), respectively, confirming the formation of hydrogels that can resist external shear force. Meanwhile, the *G*´ and *G*´´ values between **Gel Nap‐S** and **Gel Nap‐S+HG** did not present obvious difference, suggesting that coassembly of **HG** did not affect the mechanical and viscoelastic properties of **Gel Nap‐S**. In addition, the relatively small *G*´ and *G*´´ values of **Gel Nap‐S** and **Gel Nap‐S+HG** suggested that the two hydrogels were soft and injectable. After that, circular dichroism (CD) was selected to investigate the secondary structures of the assemblies in the hydrogels. As displayed in Figure 1c,d, the CD spectra of **Gel Nap‐S** and **Gel Nap‐S+HG** exhibited a positive peak at 205 nm and a negative peak at 220 nm, suggesting *β*‐sheet‐like secondary structures formed in both hydrogels.^[^ ^29^ ^]^ We noted that the CD spectrum of **Gel Nap‐S** showed a positive peak at 235 nm, which should be assigned to the chiral phenylalanine residue.^[^ ^30^ ^]^ However, this peak became flat in that of **Gel Nap‐S+HG**. We hypothesized that this phenomenon may be ascribed to coassembly of **HG** affecting the phenylalanine residue packing of **Nap‐S**. Furthermore, transmission electron microscopy (TEM) observation was conducted to reveal the nanostructures in **Gel Nap‐S** and **Gel Nap‐S+HG** (Figure 1e,f). TEM images clearly showed that uniform nanofibers formed in **Gel Nap‐S** with an average diameter of 14.4 ± 3.0 nm, while entangled and dense nanofibers exhibited in **Gel Nap‐S+HG** with an average diameter of 30.6 ± 5.4 nm. The average nanofiber diameter in **Gel Nap‐S+HG** is larger than that in **Gel Nap‐S**, echoing that **HG** indeed coassembled with the phenylalanine residues of **Nap‐S**. Taken together, above results confirmed that **HG** stably coassembled with **Nap‐S** to form injectable **Gel Nap‐S+HG** for local administration of the SIK2 inhibitor.

### 2.2 SIK2‐Instructed Disassembly of Gel Nap‐S+HG and Release of HG in Vitro

**Figure 2:**
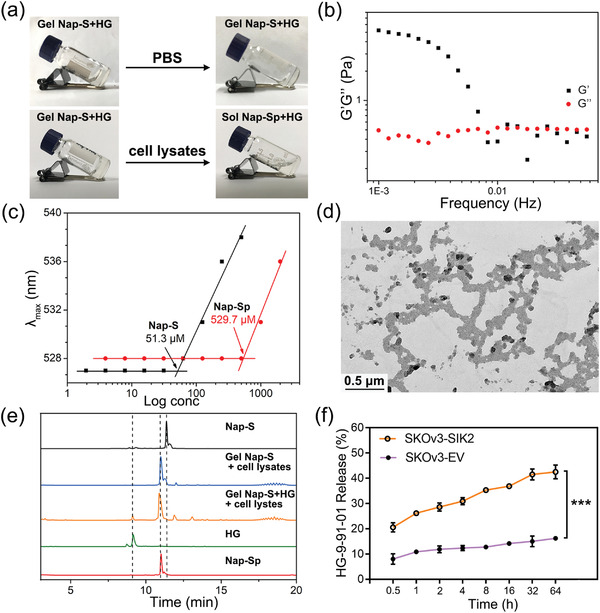
SIK2‐triggered disassembly of **Gel Nap‐S+HG** and release of **HG** in vitro. a) Optical images of 1.0 wt% **Gel Nap‐S+HG** (molar ratio: **Nap‐S**/**HG** = 125/1) before and after incubation with PBS or cell lysates overnight at 37 °C, respectively. b) Frequency dependence of the dynamic storage moduli (*G*´) and the loss moduli (*G*´´) of 1.0 wt% **Gel Nap‐S+HG** (molar ratio: **Nap‐S**/**HG** = 125/1) after incubation with cell lysates overnight (strain: 1.0%). c) CMCs for pure compounds **Nap‐S** and **Nap‐Sp**. d) TEM image of **Gel Nap‐S+HG** after incubation with cell lysates overnight. e) HPLC traces of **Gel Nap‐S** and **Gel Nap‐S+HG** after incubation with cell lysates overnight. Wavelength for detection: 280 nm. f) Cumulative release profile of **HG** in culture medium after incubation **Gel Nap‐S+HG** (i.e., 530 × 10^−6^ m for **Nap‐S**, 4.24 × 10^−6^ m for **HG**) with SKOv3‐SIK2 cells and SKOv3‐EV cells at different times, respectively. Data were presented as mean ± SD, *n* = 3. Statistical significance was assessed using one‐way ANOVA with Tukey's post‐test. ***p < 0.001.

After characterizations of **Gel Nap‐S** and **Gel Nap‐S+HG**, their SIK2‐activated gel‐to‐sol transition properties were then evaluated. Specifically, the obtained hydrogels were incubated with lysates of SKOv3‐SIK2 cells that stably overexpress SIK2 (Figure S9, Supporting Information) at 37 °C overnight, respectively. As expected, both hydrogels exhibited gel‐to‐sol transitions in response to SKOv3‐SIK2 cell lysates (**Figure** **2**a; and Figure S10, Supporting Information) while the PBS‐treated hydrogels remained in the gel state. The gel‐to‐sol transitions of these two hydrogels were further evidenced by their dynamic rheological tests (Figure 2b; and Figures S11 and S12, Supporting Information). Critical micelle concentration (CMC) measurements of the pure compounds **Nap‐S** and **Nap‐Sp** showed that their CMC values were 51.3 and 529.7 × 10^−6^ m, respectively (Figure 2c), further validated the feasibility of above gel‐to‐sol transitions. TEM analysis revealed that, after incubation with SKOv3‐SIK2 cell lysates, the original nanofibers in **Gel Nap‐S+HG** and **Gel Nap‐S** turned into uneven nanoparticles (Figure 2d; and Figure S13, Supporting Information). TEM image of pure compound **Nap‐Sp** at 1.0 wt% showed that compound self‐assembled into uniform nanoparticles with an average diameter of 94.3 ± 9.7 nm (Figure S14, Supporting Information), suggesting above uneven nanoparticles are made of the phosphorylated product of **Nap‐S** by SIK2 (i.e., **Nap‐Sp**). In addition, HPLC analyses were performed to reveal the chemical evolutions of the gelators after the hydrogels transited to solutions. The results in Figure 2e clearly indicated that most of **Nap‐S** molecules in **Gel Nap‐S** or **Gel Nap‐S+HG** was converted to their hydrophilic phosphates **Nap‐Sp** after SIK2 activation. Collectively, upon the amphiphilic hydrogelator **Nap‐S** was phosphorylated by SIK2 to yield the hydrophilic product **Nap‐Sp**, the microscopic nanofiber structures in the hydrogels transformed to nanoparticles, resulting in macroscopic gel‐to‐sol transitions.

After that, we measured the release kinetics of the SIK2 inhibitor **HG** from **Gel Nap‐S+HG** after 100 µL hydrogel was incubated with 1.0 × 10^5^ SKOv3‐SIK2 cells in 1.0 mL culture medium. At different incubation times, the culture medium was collected for HPLC analysis. Quantitative results in Figure 2f showed that **HG** in **Gel Nap‐S+HG** was released in a sustained manner. At 64 h, about 42.5% **HG** was released from the hydrogel to cell culture medium. Interestingly, about 33.8% of **HG** was uptaken by the cells. Besides, only the phosphate product **Nap‐Sp** was detected in the culture medium (Figure S15, Supporting Information), suggesting **HG** was released upon **Gel Nap‐S+HG** was phosphorylated and disassembled by SKOv3‐SIK2 cells. In addition, SKOv3‐EV cells with relatively lower SIK2 expression were also cultured in **Gel Nap‐S+HG**‐containing medium for control experiments. As shown in Figure 2f, after 64 h incubation, less than 20.0% of **HG** was released from **Gel Nap‐S+HG**, indicating excellent drug encapsulation stability of the hydrogel. These results collectively indicated that **Gel Nap‐S+HG** could be selectively disassembled by SKOv3‐SIK2 cells to release **HG** in a controllable manner.

### 2.3 Anticancer and Antimetastasis Activities of Gel Nap‐S+HG in Vitro

**Figure 3:**
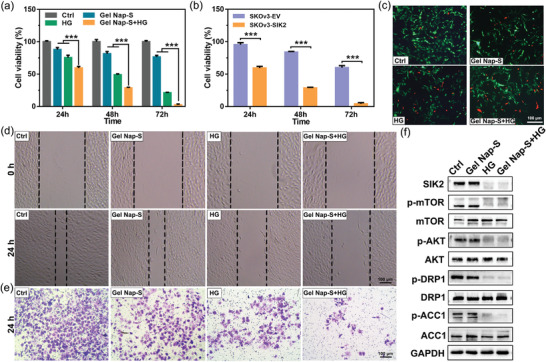
Therapeutic effects of **Gel Nap‐S+HG** in vitro. a) Cell viability of SKOv3‐SIK2 cells incubated with DMEM (Ctrl), **Gel Nap‐S** (i.e., 530 × 10^−6^ m **Nap‐S**), **HG** (4.24 × 10^−6^ m), and **Gel Nap‐S+HG** (i.e., 530 × 10^−6^ m for **Nap‐S**, 4.24 × 10^−6^ m for **HG**) at 24, 48, or 72 h. b) Cell viability of SKOv3‐SIK2 cells or SkOv3‐EV cells incubated with **Gel Nap‐S+HG** (530 × 10^−6^ m for **Nap‐S,** 4.24 × 10^−6^ m for **HG**) at 24, 48, or 72 h. Data were presented as mean ± SD, *n* = 3. Statistical significance was assessed using one‐way ANOVA with Tukey's post‐test. ***p < 0.001. c) Annexin V‐PE apoptosis assay of SKOv3‐SIK2 cells incubated with DMEM (Ctrl), **Gel Nap‐S** (530 × 10^−6^ m **Nap‐S**), **HG** (4.24 × 10^−6^ m), or **Gel Nap‐S+HG** (530 × 10^−6^ m **Nap‐S** + 4.24 × 10^−6^ m **HG**) for 24 h. Green: green fluorescent protein‐fused SIK2 (*λ*_Ex_ = 470/40 nm, *λ*_Em_ = 520/50 nm), Red: Annexin V‐PE (*λ*_Ex_ = 546/12 nm, *λ*_Em_ = 575–640 nm). Scale bars: 100 µm. d) Wound healing and e) transwell matrigel invasion assays of SKOv3‐SIK2 cells with different treatments. f) Western blot results of the expression levels of SIK2 and its downstream proteins in each group.

After investigating its **HG** release profile in response to SIK2, we next studied anticancer and antimetastasis effects of **Gel Nap‐S+HG**. First, of **Gel Nap‐S**, **HG**, and **Gel Nap‐S+HG** on SKOv3‐SIK2 cells were evaluated with cell‐counting kit‐8 (CCK‐8) assay. As shown in **Figure** **3**a; and Figures S16–S18 (Supporting Information), the viability of the SKOv3‐SIK2 cells that treated with 10 µL of 1.0 wt% **Gel Nap‐S** (i.e., 530 × 10^−6^ m **Nap‐S**) or 4.24 × 10^−6^ m **HG** for 72 h decreased to 76.6% or 21.0%, respectively. In contrast, 10 µL of 1.0 wt% **Gel Nap‐S+HG** (i.e., 530 × 10^−6^ m **Nap‐S** + 4.24 × 10^−6^ m **HG**) showed a greatly enhanced ovarian cancer cell killing efficiency (cell viability: 2.4%). To evaluate whether SIK2 overexpression contribute the excellent anticancer efficacy of **Gel Nap‐S+HG**, we tested the cytotoxicity of the hydrogel on empty vector‐transduced SKOv3 (SKOv3‐EV) cells, which expresses relatively lower level of SIK2 (Figure S9, Supporting Information). As shown in Figure 1, 3 µL of 1.0 wt% **Gel Nap‐S+HG** exhibited a significantly lower cytotoxicity (*p* <***) on SKOv3‐EV cells than on SKOv3‐SIK2 cells. These results validate that the promoted cytotoxicity of **Gel** **Nap‐S+HG** toward ovarian cancer cells mainly originate from the SIK2 activity of the cells, which triggers the disassembly of the hydrogel and subsequent release of **HG**. To investigate whether above cytotoxicity was induced by apoptosis, Annexin V‐PE staining on the cells was performed (Figure 3c). Quantitative analysis in Figure S19 (Supporting Information) showed that the apoptotic rate in **Gel Nap‐S+HG** group was 2.8‐fold, 1.6‐fold, or 5.5‐fold of that in **Gel Nap‐S**, **HG**, or control (Ctrl) group, respectively. These results collectively suggested that SIK2‐responsive and sustained release of **HG** from **Gel Nap‐S+HG** significantly improved the anticancer efficacy of **HG** on ovarian cancer cells.

Considering that metastasis is a typical feature of ovarian cancer, we further explored whether the sustained release of **HG** from our SIK2‐responsive **Gel Nap‐S+HG** could inhibit cell metastasis by performing scratch wound healing, matrigel invasion and migration assays. The results of the scratch wound healing assay, which shows the migration ability of the cells in **Gel Nap‐S**, **HG**, **Gel Nap‐S+HG**, or control group, are shown in Figure 3d. As expected, SKOv3‐SIK2 cells in the control group exhibited a strong and aggressive wound healing ability after scratching. Quantitative analysis in Figure S20a (Supporting Information) revealed **Gel Nap‐S+HG**‐treated cells had the lowest healing rate (30.6% of that of control cells), followed by **HG**‐treated cells (61.8%) and **Gel Nap‐S**‐treated cells (92.7%). Transwell matrigel invasion assay and transwell migration assay consistently showed that the **Gel Nap‐S+HG**‐treated SKOv3‐SIK2 cells had the lowest invasion and longitudinal migration ability followed by those cells in **HG**, **Gel Nap‐S**, and control groups (Figure 3e; and Figures S20b and S21, Supporting Information). All these results indicated that the sustained release of **HG** from **Gel Nap‐S+HG** significantly enhanced its suppression effects on SKOv3‐SIK2 cell migration and invasion.

Previous studies demonstrated that SIK2 plays an important role in ovarian cancer cell metabolism via several signaling pathways^22^. Therefore, after above different treatments, the SKOv3‐SIK2 cells were lysed and western blot assay was performed to detect the expression levels of SIK2 and its downstream proteins in these cells. As shown in Figure 3f; and Figure S22a (Supporting Information), **Gel Nap‐S+HG**‐treated cells had the lowest expression level of SIK2 (34.1% of that of control cells), followed by **HG**‐treated cells (57.7%) and **Gel Nap‐S**‐treated cells (98.8%). We noted that, although SIK2 in **Gel Nap‐S+HG**‐treated cells were not directly inhibited by free **HG** such as that in **HG**‐treated cells, the former was more inhibited than the latter, suggesting that sustained release of **HG** had better inhibitory effect on SIK2 than free **HG**. Consequently and as expected, phosphorylation of SIK2 downstream proteins (e.g., AKT, mTOR, DRP1, and ACC1) was inhibited at the largest extent in **Gel Nap‐S+HG**‐treated cells among all four groups studied (Figure S22b–e, Supporting Information). Above results conclusively suggested that, compared with free **HG**, sustained release of **HG** from **Gel Nap‐S+HG** could more effectively down‐regulate the expression of SIK2, as well as suppress the phosphorylation of its downstream proteins. Thus, enhanced suppression effect on ovarian cancer proliferation and metastasis might be achieved by **Gel Nap‐S+HG**.

### 2.4 Inhibitory Effect of Gel Nap‐S+HG on Ovarian Tumor Metastasis in Vivo

**Figure 4:**
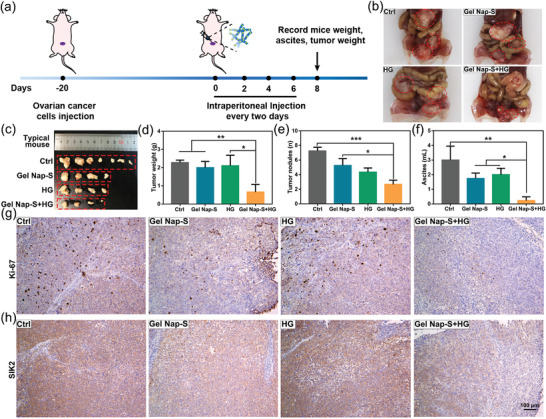
Tumor proliferation and metastasis inhibition effects of **Gel Nap‐S+HG** in vivo. a) Schedule of the establishment of peritoneal metastasis SKOv3‐SIK2 ovarian cancer mouse models (*n* = 6 per group), i.p. injection of **Gel Nap‐S+HG**, tumor measurements, and analyses. b,c) Representative images of omental metastasis tumors (red circles) after i.p. injected with PBS, **Gel Nap‐S**, **HG**, or **Gel Nap‐S+HG**, then all mice in four groups were sacrificed at day 8. d) Tumor weight and e) tumor nodules, and f) ascites volume of all mice in four groups were recorded after the sacrificed. Data were presented as mean ± SD, *n* = 6. Statistical significance was assessed using one‐way ANOVA with Tukey's post‐test. *p < 0.05; **p < 0.01; ***p < 0.001. Representative immunohistochemical staining of Ki‐67 g) and SIK2 h) of tumors in four groups at day 8.

Encouraged by above positive results, we subsequently investigated whether **Gel Nap‐S+HG** could additionally enhance the inhibitory effect of **HG** on SKOv3‐SIK2 tumor metastasis in vivo. Since advanced ovarian cancer usually metastasizes to its adjacent omentum tissues, SKOv3‐SIK2 cells were intraperitoneally injected into Balb/c nude mice to build up the peritoneal metastasis ovarian cancer mouse model to evaluate the in vivo efficacy of **Gel Nap‐S+HG**. 20 days after SKOv3‐SIK2 cells injection, mice were randomly selected for whole‐body fluorescence imaging first. As shown in Figure S23 (Supporting Information), no obvious fluorescence was observed from the mice before dissection. However, after the mice were dissected, bright green fluorescence was observed from the metastasis foci on omentum and mesentery, indicating the successful establishment of peritoneal metastasis ovarian cancer models in mice (Figure S24, Supporting Information). As illustrated in **Figure** **4**a, **Gel Nap‐S**, **HG**, or **Gel Nap‐S+HG** was intraperitoneally (i.p.) injected into the peritoneal metastasis mice at Nap‐S dose of 117.65 mg kg^−1^ or **HG** dose of 0.28 mg kg^−1^ every 2 days, respectively. In addition, a group of PBS‐treated mice was designated as a control group. At day 8, we found that one peritoneal metastasis mouse in the control group died, and the rests displayed markedly reduced food intake and activity. Therefore, on day 8 after treatment, all the experimental mice were sacrificed for further analyses. As shown in Figure 4b,c, in both **Gel Nap‐S** and **HG**‐treated groups, the metastatic tumors on omentum and mesentery showed consistent growth rate with that in PBS group. In contrast, in **Gel Nap‐S+HG**‐treated group, average volume of the metastatic tumors was obviously smaller than those in other three groups, indicating the most inhibitory capacity of **Gel Nap‐S+HG** on ovarian tumor metastasis. Further measurements of tumor weight, number of metastatic nodules, and ascites volume in these four groups additionally confirmed the best inhibitory effect of **Gel Nap‐S+HG** on ovarian tumor metastasis. The quantitative results in Figure 4d–f showed that after **Gel Nap‐S+HG** treatment, above three indicators were reduced to 28.8%, 36.8%, and 7.8% of those of PBS‐control group, respectively. After that, we performed Ki67 and immunohistochemical (IHC) staining on the metastatic tumors. As shown in Figure 4g, proliferation of the tumors in **Gel Nap‐S+HG**‐treated group was the most significantly inhibited. Consistently, as shown in Figure 4h, compared with those in other three groups, expression level of SIK2 in **Gel Nap‐S+HG**‐treated remarkably decreased. The more efficient down‐regulation of SIK2 in **Gel Nap‐S+HG** group than **HG** group could be explained as that **HG** was responsively and sustained released in the former group, whereas in the latter group **HG** was directly administered and degraded quickly in vivo^27^. These results clearly indicated that our SIK2‐responsive **Gel Nap‐S+HG** has excellent capacity of SIK2 inhibition and consequent ovarian tumor metastasis suppression in vivo. Moreover, body weights of all mice were monitored to assess the side effects of these treatments. As shown in Figure S25 in the Supporting Information, no significant weight loss was observed in all group mice during the observation times. To further evaluate the pathological effect of **Gel Nap‐S+HG**, **HG**, and **Gel Nap‐S** on the mice, after treatments, major organs such as the heart, liver, spleen, lung, and kidney were taken from the mice and sliced for hematoxylin and eosin (H&E) staining. The results in Figure S26 (Supporting Information) revealed that none of the treatments impose obvious pathological change on the major organs. Collectively, besides its best inhibitory effect on ovarian tumor metastasis, **Gel Nap‐S+HG** also displayed good biosafety to the mice.

## 3 Conclusion

In summary, to improve the inhibitory efficacy of a SIK2 inhibitor **HG** on ovarian tumor growth and metastasis, we rationally designed a hydrogelator **Nap‐S** and coassembled **HG** with the hydrogelator to form a hydrogel **Gel Nap‐S+HG**. Upon local administration of **Gel Nap‐S+HG**, **HG** was SIK2‐responsive released from the hydrogel, exhibiting an enhanced inhibitory effect on ovarian tumor growth and metastasis. Hydrogelation and rheology experiments showed that **HG** could coassemble with **Nap‐S** to form a stable and transparent hydrogel suitable for peritoneal injection. CD spectra of the hydrogels **Gel Nap‐S+HG** indicated that **HG** coassembled with the phenylalanine residues of **Nap‐S** but did not affect the *β*‐sheet secondary structures of the hydrogelator in the hydrogel. TEM images of the hydrogels showed that the coassembly of **HG** with **Nap‐S** resulted in entangled and denser nanofibers in the hydrogel. These characterization results revealed that the **HG** indeed coassembled (but not physically mixed) well with **Nap‐S** in the supramolecular hydrogel. In vitro experiments indicated that, upon overnight incubation with SIK2‐overexpressing ovarian cancer cell lysates, **Gel Nap‐S+HG** underwent a gel‐to‐sol transition, accompanied by **HG** release. Further HPLC analysis revealed that the disassembly of the hydrogel was attributed to the efficient enzymatic conversion of **Nap‐S** to **Nap‐Sp** by SIK2. Cumulative release profile suggested that **HG** was released from **Gel Nap‐S+HG** in a sustainable manner up to 64 h. Cell experiments validated that the sustained release of **HG** from **Gel Nap‐S+HG** significantly improved its antiproliferation and antimetastasis activities against ovarian cancer cells. Further western blot assay revealed that **Gel Nap‐S+HG**‐treatment significantly down‐regulated the expression of SIK2 and consequently inhibited the phosphorylation of its downstream signaling proteins (e.g., AKT/mTOR, DRP1, and ACC1). To additionally test its inhibitory effect of **Gel Nap‐S+HG** on tumor metastasis in vivo, peritoneal metastasis SKOv3‐SIK2 ovarian cancer mouse models were established, followed by i.p. injection of the hydrogel and controls. As expected, compared with free **HG** and **Gel Nap‐S**, **Gel Nap‐S+HG** exhibited stronger inhibitory effects on ovarian tumor growth and metastasis, as evidenced by the obviously decreased tumor weight, number of metastatic nodules, and ascites volume in **Gel Nap‐S+HG** group. IHC staining echoed that SIK2 expression in ovarian cancer cells was suppressed at the largest extent in **Gel Nap‐S+HG** group. Collectively, our **Gel Nap‐S+HG** exhibited an excellent capacity of suppression on ovarian cancer proliferation and metastasis via SIK2 inhibition both in vitro and in vivo. Recently, some hydrogel drug delivery systems have been approved for clinical treatment.^[^ ^21b^ ^]^ A typical example is VANTAS hydrogel implants, which can continuously deliver histrelin for prostate cancer therapy.^[^ ^31^ ^]^ In addition, we confirmed that SIK2 is overexpressed in the ovarian cancer cells from human patient tumor tissues (Figure S27, Supporting Information). Encouraging by these positive news, we anticipate that our hydrogel **Gel Nap‐S+HG** might be translated and applied for ovarian cancer therapy in clinic in the near future.

## 4 Experimental Section

### Evaluation of Therapeutic Effect In Vivo

All experimental operations were performed with the consent of the Animal Care Committee of Southeast University (No.20211116004) and complied with the Regulations for the Administration of Affairs Concerning Experimental Animals of China. To evaluate the therapeutic effect of **Gel Nap‐S**, **HG**, and **Gel Nap‐S+HG**, peritoneal metastasis mice models were established. First of all, SKOv3‐SIK2 cells were intraperitoneally (i.p.) injected into 5‐weeks‐old Balb/c nude mice (17 ± 1 g, Yangzhou University Medical Center, China) at the density of 4 × 10^7^ cells mL^−1^. 20 days later, mice were randomly divided into 4 groups: 1) i.p. injected with PBS, 2) i.p. injected with **Gel Nap‐S** (hydrogelator dose: 117.65 mg kg^−1^), 3) i.p. injected with **HG** (inhibitor dose: 0.28 mg kg^−1^), 4) i.p. injected with **Gel Nap‐S+HG** (hydrogelator dose: 117.65 mg kg^−1^ + inhibitor dose: 0.28 mg kg^−1^). All these treatments were given every two days and during the whole time, mice body weight was recorded. 8 days later, mice were sacrificed and tumor nodules, weight, and ascites were recorded. Metastasis tumors were dissected for immunohistochemical (IHC) staining and important organs were collected for H&E staining.

### Tissue Staining

For IHC staining, tumor sections in each group (Ctrl, **Gel Nap‐S**, **HG**, and **Gel Nap‐S+HG**) were deparaffinized an d hydrated following the standard protocol. Then antigen retrieval was conducted by incubating the sections with boiling water for 20 min. Afterward, tumor sections were incubated with rabbit anti‐SIK2 (Cell signaling) and Ki‐67 (Abcam, ab279653) at 4 °C overnight. Color was developed using diaminobenzidine (DAB) substrate followed by hematoxylin counter staining. For H&E staining, mice important organs like hearts, livers, spleens, lungs, and kidneys were fixed by 4% paraformaldehyde solutions. Then tissues were embedded in paraffin and sectioned. Afterward, all these sections were stained by hematoxylin and eosin following the standard protocol.

### Ovarian Cancer Tumor Tissue Samples Collection

Ovarian cancer tumor tissues (3 for western blot analysis) were obtained from Biobank of Zhongda Hospital of Southeast University. This study was performed with the approval of the Ethnic Committee of Zhongda Hospital (2020ZDSYLL307‐P01/2021.02.03).

### Statistical Analysis

Data values were presented as mean ± standard deviation (SD). All experiments were repeated at least three times, and quantified with at least triplicates. Student's *t*‐test or one‐way ANOVA followed by Tukey correction were used to compare the differences between groups. *P* value < 0.05 is considered statistically significant (*p < 0.05, **p < 0.01, ***p < 0.001). All statistical tests were calculated using GraphPad Prism.

## Conflict of Interest

The authors declare no conflict of interest.

## Supporting information

Supporting Information

## Data Availability

The data that support the findings of this study are available from the corresponding author upon reasonable request.
