# Supplementary material for: Salt‐Inducible Kinase 2‐Triggered Release of Its Inhibitor from Hydrogel to Suppress Ovarian Cancer Metastasis
Source: Adv Sci (Weinh). 2022 May 26;9(22):2202260. doi: 10.1002/advs.202202260 (PMC9353504; doi:10.1002/advs.202202260)
Supplement: Supplementary file 1 — Supporting Information [file ADVS-9-2202260-s001.pdf]

ADVANCED SCIENCE
Open Access

Supporting Information

for *Adv. Sci.*, DOI 10.1002/advs.202202260

Salt-Inducible Kinase 2-Triggered Release of Its Inhibitor from Hydrogel to Suppress Ovarian Cancer Metastasis

Yue Hua, Han Yin, Xiaoyang Liu, Jinbing Xie, Wenjun Zhan, Gaolin Liang* and Yang Shen**

Supporting Information

Salt-inducible Kinase 2-Triggered Release of Its Inhibitor from Hydrogel to Suppress Ovarian Cancer Metastasis

Yue Hua, Han Yin, Xiaoyang Liu, Jinbing Xie, Wenjun Zhan, Gaolin Liang* and Yang Shen**

Y. Hua, H. Yin, Prof. J. Xie, Prof. Y. Shen
Department of Obstetrics and Gynaecology, Zhongda Hospital, School of Medicine, Southeast University, Nanjing, Jiangsu 210009, China
E-mail: shenyang@seu.edu.cn
X. Liu, Dr. W. Zhan, Prof. G. Liang
State Key Laboratory of Bioelectronics, School of Biological Science and Medical Engineering, Southeast University, 2 Sipailou Road, Nanjing 210096, China
E-mail: wjzhan@seu.edu.cn; gliang@seu.edu.cn

This file includes:

1. General methods
2. Syntheses of **Nap-S** and **Nap-Sp** (**Scheme S1**)
3. Supporting figures (**Figure S1-S27, Table S1**)

1. General methods

Experimental Materials and Instruments

2-Chlorotrityl chloride resin, Fmoc protected amino acid, O-benzotriazole-N,N,N',N'-tetramethyluronium-hexafluorophosphate (HBTU), 1-hydroxybenzotriazole (HOBt) were obtained from GL Biochem Co., Ltd. (Shanghai). N, N-diisopropylethylamine (DIPEA) and trifluoroacetic acid (TFA) was purchased from Sigma-Aldrich (Shanghai, China). Commercially available reagents were used without further purification. All other chemicals were reagent grade or better. Deionized (DI) water (18.2 MΩ·cm) was purified by a Milli-Q system (Millipore) and used throughout the experiments. High performance liquid chromatography (HPLC) purification was performed on a Shimazu UFLC system equipped with two LC-20AP pumps and an SPD-20A UV/vis detector using a Shimazu PRC-ODS column. HPLC analyses were conducted on an Agilent 1260 HPLC system equipped with a G1322A pump and in-line diode array UV detector using an Agilent Zorbax 300SB-C18 RP column with CH_3CN (0.1% of TFA) and ultrapure water (0.1% of TFA) as the eluent. Electrospray ionization-mass spectrometry (ESI-MS) test was performed on a Finnigan LCQ Advantage ion trap mass spectrometer (ThermoFisher Corporation) that was equipped with a standard ESI source. ^{1}H NMR and ^{13}C NMR spectrum were recorded on a Bruker AV 300. Circular dichroism (CD) spectra were obtained on a Chriascan qCD (Applied Photophysics, England). Rheology test was conducted by Haake RheoStress 6000 (Thermo Scientific), with cone-and plate geometry (1°/20 mm) at the gap of 370 µm.

TEM Sample Preparation

The corresponding samples were dropped on carbon-coated grids and air-dried overnight. Then the TEM images were obtained with a JEM-2100 transmission electron microscope (JEOL, Japan).

CMC Experiment

Nap-S and **Nap-Sp** solutions at concentrations of 2 mM to 1.95 µM were prepared in PBS (10 mM, pH 7.4). After the solutions were incubated with Rhodamine 6G (5 µM), their λmax were determined by measuring the absorbance from 520 to 540 nm using a microplate reader.

Cell Culture

Human ovarian cancer cell line SKOv3 was obtained from the American Type Culture Collection (ATCC) and maintained in Dulbecco’s Modified Eagle Medium (DMEM). Cells were cultured in a humidified incubator at 37 °C and in an atmosphere of 5% CO2 and supplemented with 10% fetal bovine serum (Gibco), 100 U/mL penicillin/streptomycin.

Ovarian cancer tumor tissue samples collection

Ovarian cancer tumor tissues (3 for western blot analysis) were obtained from Biobank of Zhongda Hospital of Southeast University. This study was performed with the approval of the Ethnic Committee of Zhongda Hospital (2020ZDSYLL307-P01/2021.02.03).

Lentivirus-Mediated Stable Cell Lines Generation

SKOv3 cells were transfected with SIK2 overexpressing vector and empty vector (Genechem, Shanghai, China) following the manufacturer’s protocols. SKOv3-SIK2 and SKOv3-EV cell lines were cultured with 10% fetal bovine serum (Gibco), and 50 ng/µL puromycin for a minimum of three passages prior to use.

Quantitative Real-time PCR

Total RNA was extracted using Trizol reagent (Invitrogen, USA) and reverse transcribed using StarScript II First-strand cDNA Synthesis Kit-II (GenStar, A214-02, China) following the manufacturer's protocols. Quantitative real-time was performed using a SYBR Green PCR kit (GeneStar, A303-10, China) in a 20 µL qPCR reaction according to the manufacturer's protocols. Each sample was tested in triplicate. β-actin was used as an internal control for all samples. Primer sequences are listed in the table below.

Primer set name	Forward Sequence	Reverse Sequence
SIK2	cagcagctgcaggaacatag	gacttggctgtgggtaggag
β-actin	tcgcctttgccgatccg	atgatctgggtcatcttcycg

SIK2-Instrusted Disassembly of Hydrogels in Vitro

100 µL SKOv3-SIK2 cell lysates (4×10^5 cells) were added an equal volume of **Gel Nap-S** and **Gel Nap-S+HG** at 37°C. After incubation overnight, **Gel Nap-S** and **Gel Nap-S+HG** were partially disassembled by SIK2 in the cell lysates.

CCK-8 Assay

SKOv3-SIK2 cells were seeded in 96-well plate (2,000 cells/well). Culture media (Ctrl group), **Gel Nap-S** (530 µM **Nap-S**), **HG** (4.24 µM) or **Gel Nap-S+HG** (530 µM **Nap-S** + 4.24 µM **HG**) was added into the plates, respectively. At day 1, 2 and 3, cell proliferation was detected using the CCK-8 assay according to the manufacturer's protocols. Briefly, the culture medium in each well was replaced with 100 µL DMEM containing 10 µL CCK-8. After incubation for 1 h at 37 °C, optical density (OD) values of absorbance at 450 nm were measured in a microplate reader (SpectraMax iD3, Molecular Devices). Each of the experiments was performed at least three times.

Cell Apoptosis Assays

As SKOv3-SIK2 cells were carried with GFP-tag, which could interfere with the green fluorescent signal, Annexin V-PE Apoptosis Detection Kit (Beyotime, C1065M, China) was added to testify the apoptotic cells at different treatments (DMEM**, Nap-S, HG** and **Nap-S+HG**). After incubation for 24h, apoptotic SKOv3-SIK2 cells were stained red and observed with fluorescence microscope (Nicon, Japan).

In Vitro Migration and Invasive Assays

To detect the migration and invasive capacity of SKOv3-SIK2 cells after different treatment, the scratch wound healing and transwell invasive assays were performed. For wound healing assays, SKOv3-SIK2 cells were seeded in a 6-well plate (2×10^4 cells/well) and then incubated with DMEM, **Gel Nap-S** (530 µM **Nap-S**), **HG** (4.24 µM) and **Gel Nap-S+HG** (530 µM **Nap-S** + 4.24 µM **HG**) for 24 h. A scratch with 200 µL yellow pipette tip was placed in the middle of the wells. Then the wells were gently washed with PBS to remove the cell debris. Afterwards, each well was added with incomplete culture medium and photographed at 0 h and 24 h. For transwell invasive assays, after treatment with DMEM, **Gel Nap-S**, **HG** and **Gel Nap-S+HG** for 24 h, SKOv3-SIK2 cells in each group were digested with trypsin and then seeded to the upper chamber at the density of 2×10^4/100 µL (dispersed in FBS-free DMEM). The lower chamber was added with 550 µL DMEM containing 20% FBS. After 24 h incubation, the chambers were washed with PBS from upper to lower twice gently, and then the migrated cells in the lower chambers were fixed with 70% (v/v) ethanol solutions for 30 min. Then the chambers were stained with 0.1% (w/v) crystal violet solution for 30 min. Later, the cells in the upper chamber were gently wiped off with a cotton swab soaked in PBS. Migrated cells were observed with microscope (XD-202, Nanjing Jiangnan Yongxin Optical

Co, Ltd.). Next, the crystal violet was eluted by a 33% acetic acid solution and measured at the absorbance of 570 nm.

Western Blot Analyses

SKOv3-SIK2 cells were cultured with DMEM, **Gel Nap-S** (**Nap-S**: 530 µM), **HG** (4.24 µM), **Gel Nap-S+HG** (530 µM **Nap-S** + 4.24 µM **HG**) and complete medium only for 24 hours. Afterwards, total proteins were extracted from the cells and separated by electrophoresis using 6.5%-10% SDS-PAGE gels, then transferred onto PVDF membranes (Millipore, USA). The membranes were incubated with specific primary antibodies overnight at 4 °C and HRP-conjugated second antibody for 1 hour at room temperature. GAPDH was used as a loading control. Proteins were visualized using an enhanced chemiluminescence system (Millipore, USA). The antibodies used and their dilutions were listed in the table below.

Antibody	Company (Cat. No.)	Working Concentration Dilutions
SIK2	Cell Signaling (#6919)	WB: 1/1000
p-Drp1(S616)	Cell Signaling (#4494)	WB: 1/1000
Drp1	Abcam (ab184247)	WB: 1/1000
p-AKT	Cell Signaling (#4060)	WB: 1/2000
AKT	Abcam (ab8805)	WB: 1/1000
p-mTOR	Cell Signaling (#5536)	WB: 1/1000
mTOR	Proteintech (66888-1-lg)	WB: 1/5000
p-ACC1	Cell Signaling (#11818)	WB: 1/1000
ACC1	Cell Signaling (#4190)	WB: 1/1000
GAPDH	Abclonal (A19056)	WB: 1/1000

2. Syntheses of Nap-S and Nap-Sp

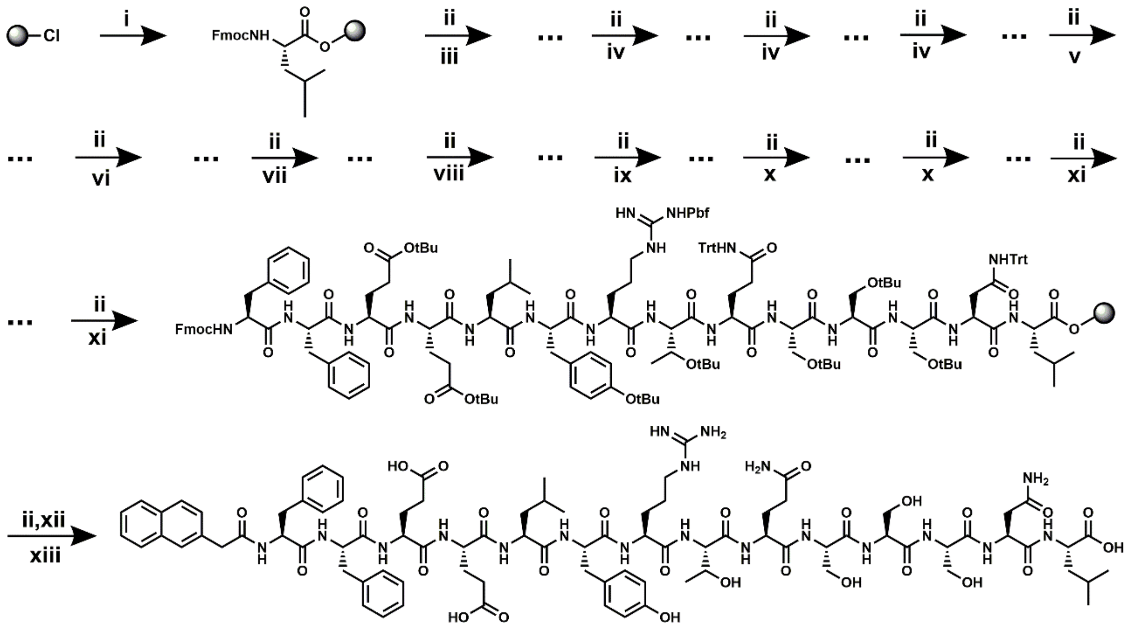

i) Fmoc-Leu-OH, DIPEA; ii) 20% piperidine; iii) Fmoc-Asn(Trt)-OH, HBTU, DIPEA; iv) Fmoc-Ser(OtBu)-OH, HBTU, DIPEA; v) Fmoc-Gln(Trt)-OH, HBTU, DIPEA; vi) Fmoc-Trt(OtBu)-OH, HBTU, DIPEA; vii) Fmoc-Arg(Pbf)-OH, HBTU, DIPEA; viii) Fmoc-Tyr(Boc)-OH, HBTU, DIPEA; ix) Fmoc-Leu-OH, HBTU, DIPEA;x) Fmoc-Gln(OtBu)-OH, HBTU, DIPEA; xi)Fmoc-Phe-OH, HBTU, DIPEA; xii) 2-Naphthaleneacetic acid, HBTU, DIPEA; xiii) 95% TFA, 2.5% H2O, 2.5% TIS

Scheme S1. The synthetic route for **Nap-S**.

Nap-S and **Nap-Sp** were synthesized with solid phase peptide synthesis (SPPS). Scheme S1 shows the synthetic route for **Nap-S**. The synthetic route for **Nap-Sp** is similar with that of **Nap-S**. The compounds were purified with HPLC. ^{1}H NMR spectrum of **Nap-S** (300 MHz, DMSO-d_6) δ : 8.12 – 7.72 (m, 14 H), 7.41 (p, J = 6.7 Hz, 3 H), 7.23 – 7.04 (m, 16 H), 6.94 – 6.57 (m, 4 H), 4.98 (t, J = 40.2 Hz, 2 H), 4.40 – 3.94 (m, 13 H), 3.54 (s, 6 H), 3.51 – 3.36 (m, 6 H), 2.99 (dd, J = 38.3, 28.4 Hz, 4 H), 2.67 (dd, J = 25.2, 15.0 Hz, 2 H), 2.15 (d, J = 46.1 Hz, 4 H), 1.93 – 1.28 (m, 18 H), 1.00 (d, J = 6.2 Hz, 3 H), 0.75 (dd, J = 24.7, 6.4 Hz, 12 H). ^{13}C NMR spectrum of Nap-S (300 MHz, DMSO-d_6) δ : 174.56, 174.44, 174.38, 174.11, 172.23, 172.10, 171.84, 171.74, 171.64, 171.60, 171.40, 171.36, 171.26, 171.19, 170.55, 170.52, 170.31, 170.24, 170.11, 157.18, 156.22, 139.51, 138.17, 138.03, 137.00, 136.83, 136.57, 134.29, 134.07, 133.68, 133.37, 132.98, 132.18, 130.50, 129.65, 128.48, 128.34, 128.02, 127.89, 127.87, 127.79, 127.68, 126.70, 126.57, 126.41, 125.89, 115.33, 67.01, 66.69, 62.18, 61.97, 58.41, 57.70, 55.52, 55.29, 54.22, 54.11, 52.62, 52.55, 52.37, 52.24, 51.56, 50.87, 50.66, 50.10, 44.31, 42.66, 42.59, 40.88, 37.84, 37.71, 37.26, 37.02, 31.78, 30.59, 30.53, 29.99, 29.55, 28.53, 27.96, 27.60, 25.35, 24.59, 24.52, 23.46, 23.31, 21.83, 20.09. MS of **Nap-S**: calcd. $[M+H]^+$: 1888.9; found ESI-MS (m/z) $[M+H]^+$: 1888.6. MS of **Nap-Sp**: calcd. $[M+H]^+$: 1968.8, $[M+K]^+$: 2006.8; found ESI-MS (m/z) $[M+H]^+$: 1969.2, $[M+K]^+$: m/z 2007.1.

3. Supporting figures and table

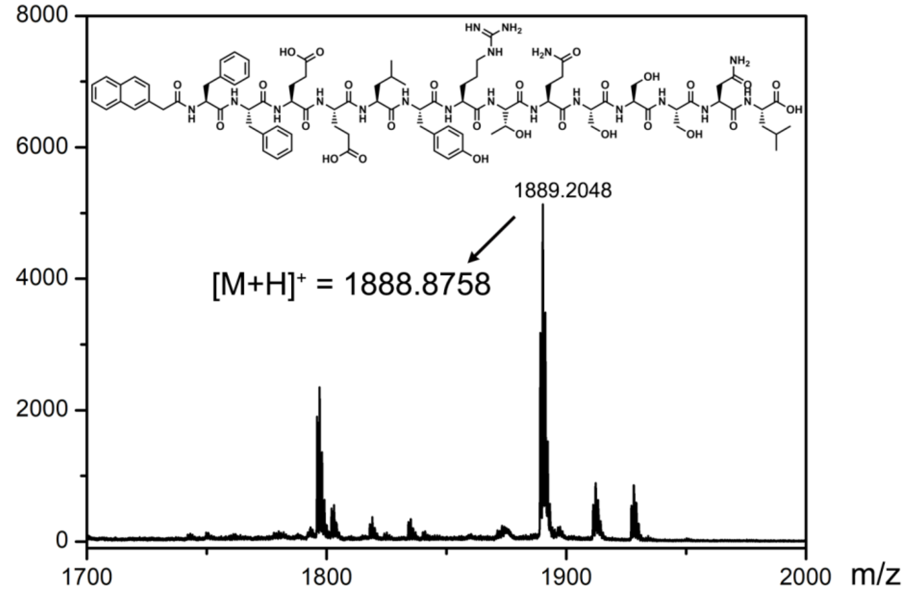

Figure S1. MALDI-TOF mass spectrum of **Nap-S**.

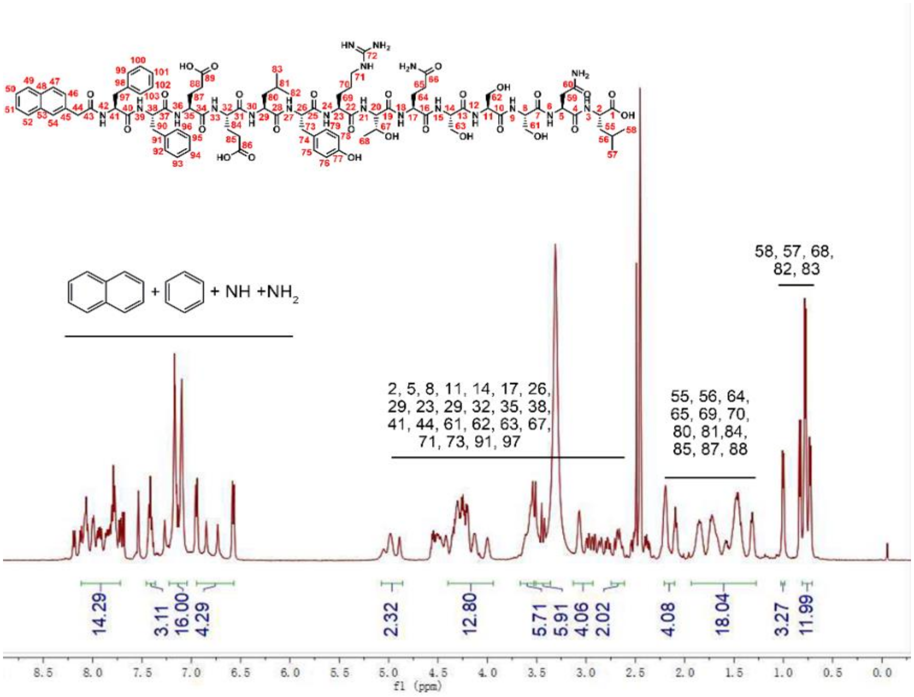

Figure S2. ^{1}H NMR spectrum of **Nap-S** in DMSO-d_6.

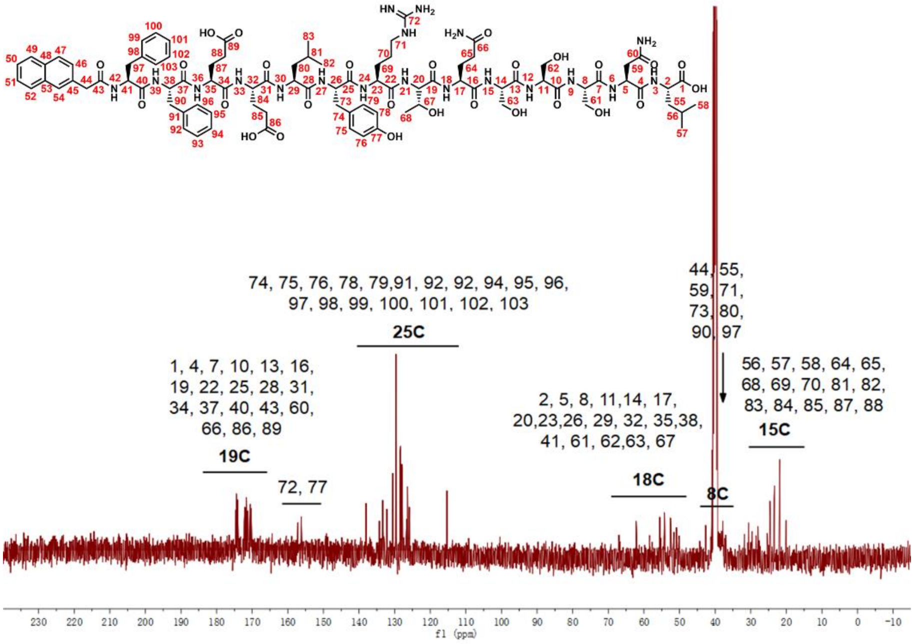

Figure S3. ^{13}C NMR spectrum of **Nap-S** in DMSO-d_6.

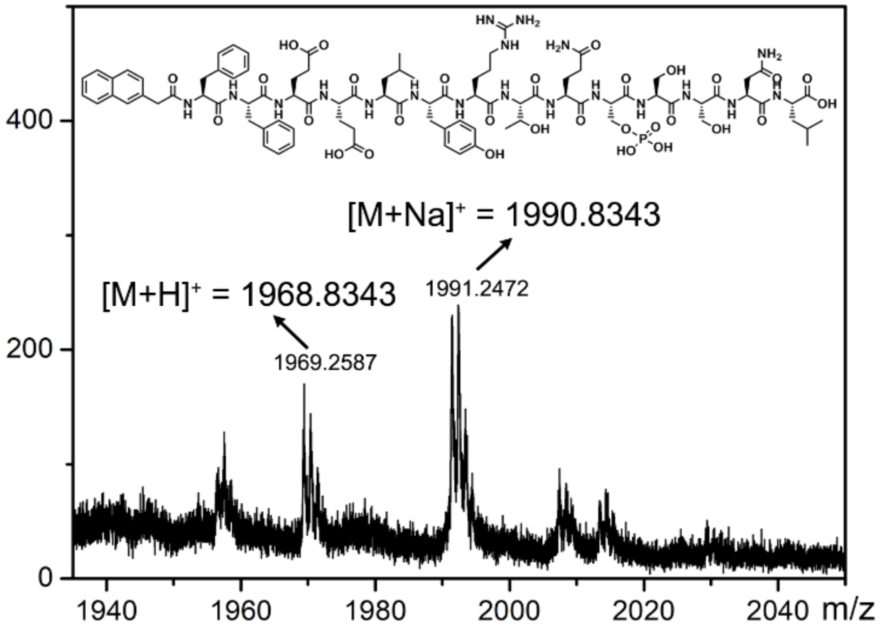

Figure S4. MALDI-TOF mass spectrum of **Nap-Sp**.

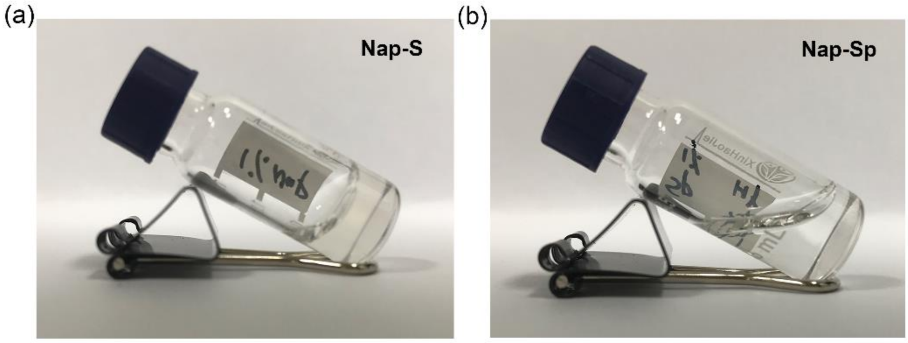

Figure S5. Optical images of 1.0 wt% (a) **Nap-S** and (b) **Nap-Sp** after heat-cooling process.

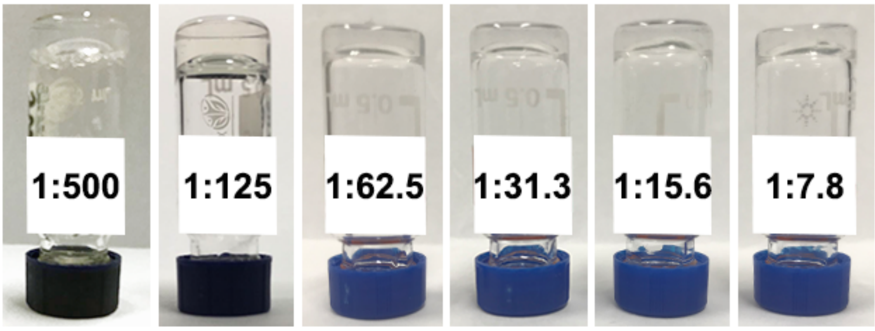

Figure S6. Optical image of 1.0 wt% **Nap-S+HG** mixtures at different **HG**/**Nap-S** molar ratios (1:500, 1:250, 1:125, 1:62.5, 1:31.3, 1:15.6, and 1:7.8) after heat-cooling process.

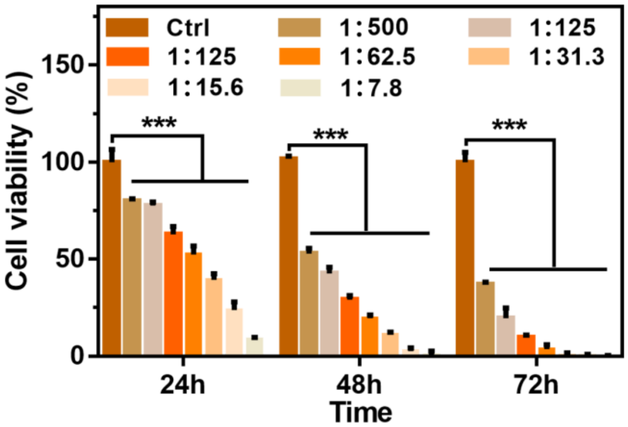

Figure S7. Cell viability assay of SKOv3-SIK2 cells incubated with co-assembled hydrogels with different **HG/Nap-S** molar ratios (1:500, 1:250, 1:125, 1:62.5, 1:31.3, 1:15.6, and 1:7.8) for 24, 48, or 72 h. Data were expressed as mean ± SD, n = 3. Statistical significance was assessed using one-way ANOVA with Tukey's post-test. *$p < 0.05$, **$p < 0.01$, ***$p < 0.001$.

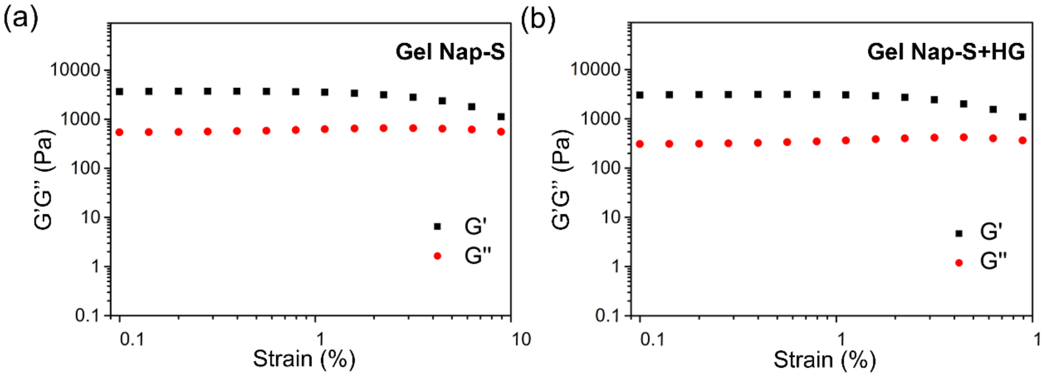

Figure S8. Strain dependence of the dynamic storage moduli (G´) and the loss moduli (G´´) of 1.0 wt% (a) **Gel Nap-S** and (b) **Gel Nap-S+HG** (molar ratio: **Nap-S/HG** = 125/1), respectively. (25 °C, frequency: 1 Hz).

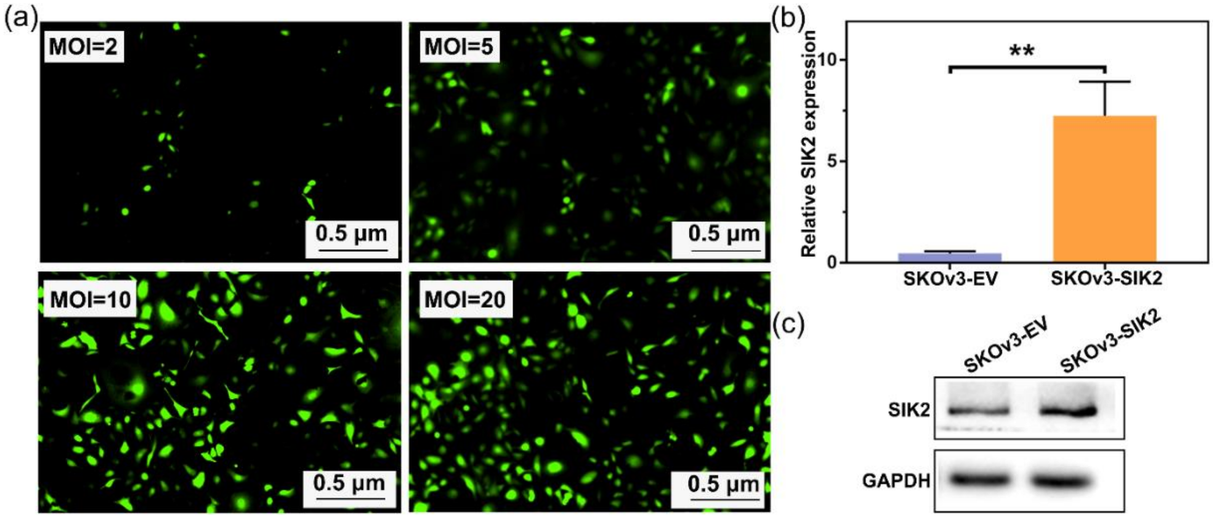

Figure S9. (a) Transfection efficiency of SKOv3 cells under MOI = 2, MOI = 5, MOI = 10, and MOI = 20. (b) SKOv3 cells were transfected with either empty virus (EV) or SIK2 overexpression virus. Expression level of SIK2 was detected by qRT-PCR. Data were expressed as mean ± SD, n = 3. Statistical significance was assessed using student's t-test.

**$p < 0.01$. (c) Western blot results of the expression levels of SIK2 in SKOv3-EV cells or SKOv3-SIK2 cells.

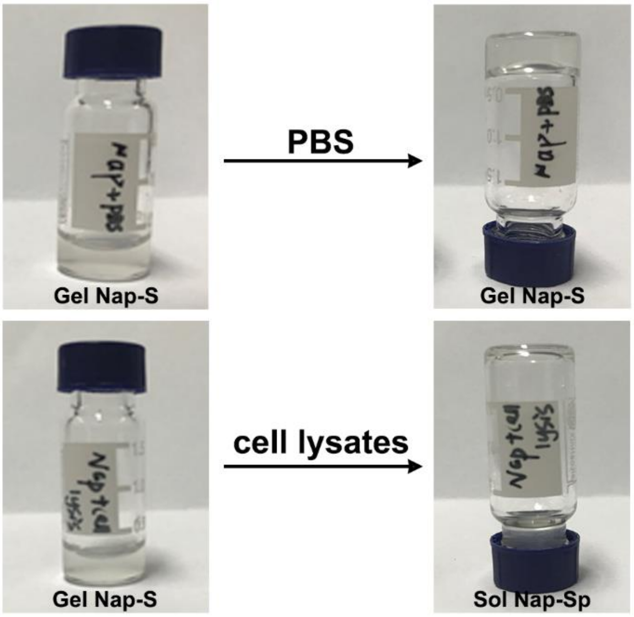

Figure S10. Optical images of 1.0 wt% **Gel Nap-S** incubated with PBS and cell lysates overnight at 37 °C, respectively.

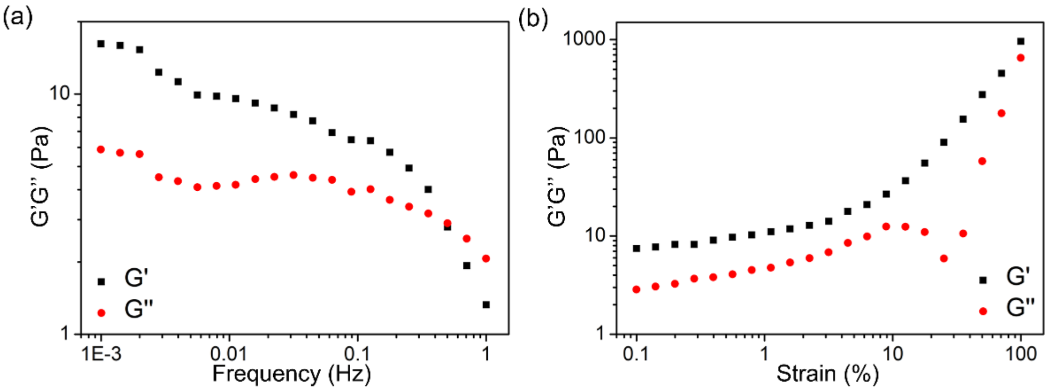

Figure S11. (a) Frequency (strain: 1.0 %) and (b) strain (frequency: 1 Hz) dependence of the dynamic storage moduli (G´) and the loss moduli (G´´) of 1.0 wt% **Gel Nap-S** after incubation with cell lysates overnight.

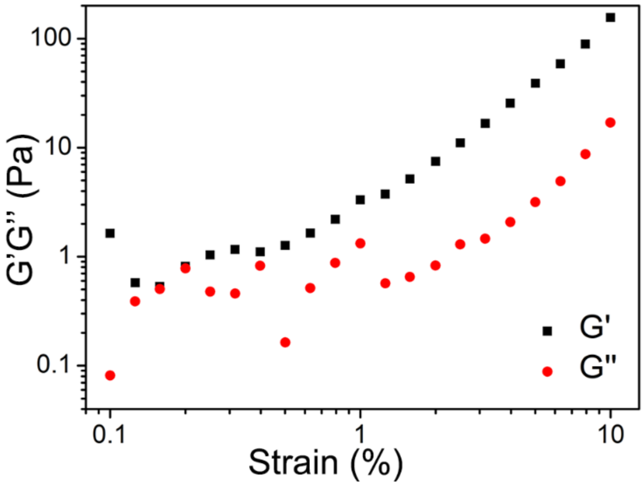

Figure S12. Strain dependence of the dynamic storage moduli (G´) and the loss moduli (G´´) of 1.0 wt% **Gel Nap-S+HG** (molar ratio: **Nap-S**/**HG** = 125/1) after incubation with cell lysates overnight (frequency: 1 Hz).

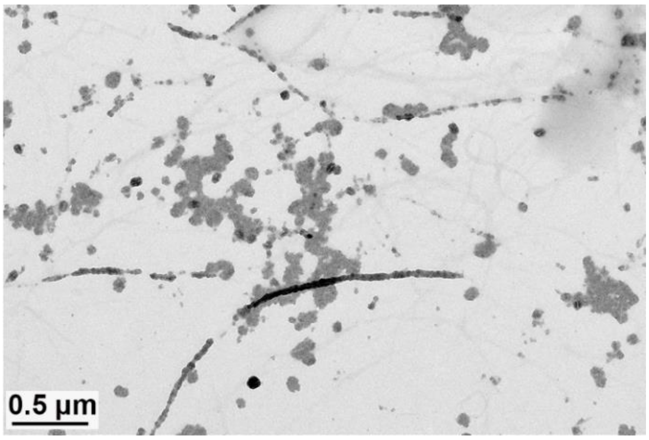

Figure S13. TEM image of 1.0 wt% **Gel Nap-S** after incubation with cell lysates overnight.

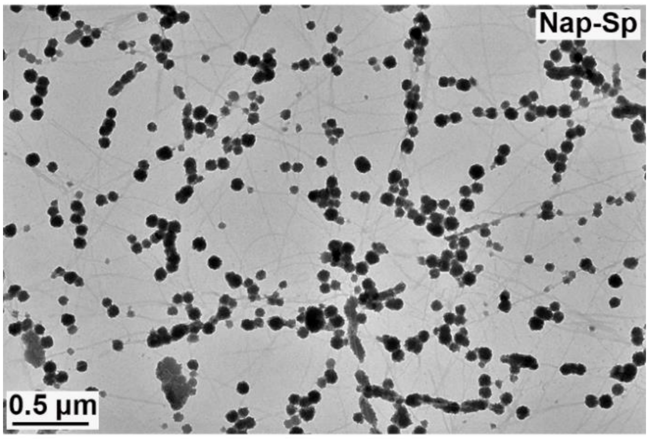

Figure S14. TEM image of 1.0 wt% **Nap-Sp** after heat-cooling process.

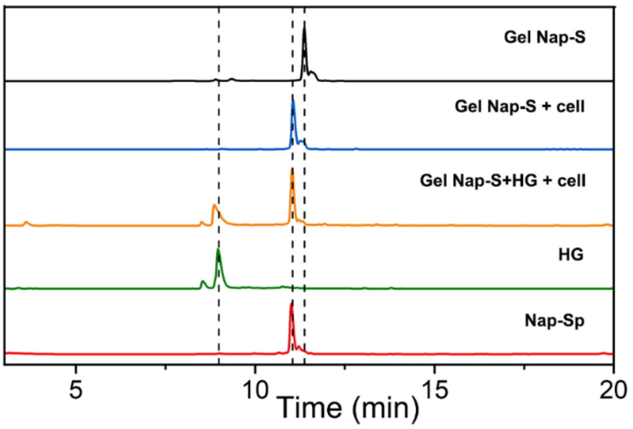

Figure S15. Culture medium in **Gel Nap-S** group and **Gel Nap-S+HG** group were collected after incubation with SKOv3-SIK2 cell for 64 h. HPLC traces of these culture medium were detected at the wavelength of 280 nm. **Nap-S, HG**, and **Nap-Sp** were detected as well, respectively.

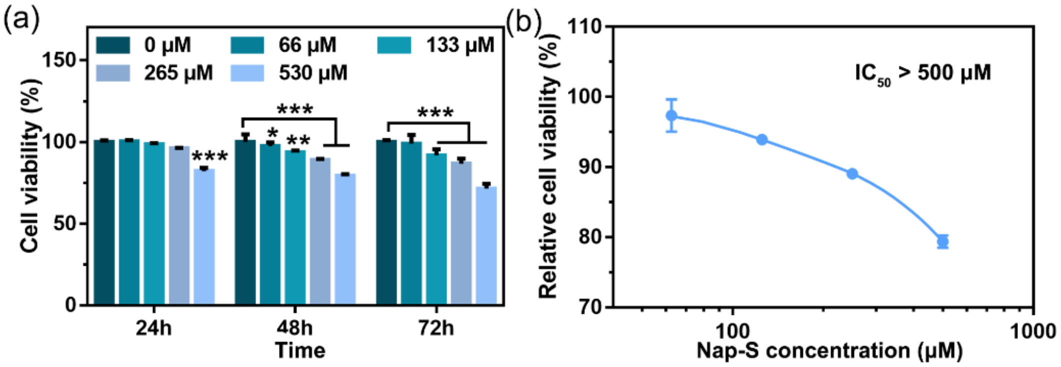

Figure S16. (a) Cell viability assay of SKOv3-SIK2 cells incubated with **Gel Nap-S** (**Nap-S**: 0-530 μM) for 24, 48, or 72 h. (b) Cell viability values at different **Nap-S** concentrations at 48 h in a. Data were expressed as mean ± SD, n = 3. Statistical significance was assessed using one-way ANOVA with Tukey's post-test. $*p < 0.05$, $**p < 0.01$, $***p < 0.001$.

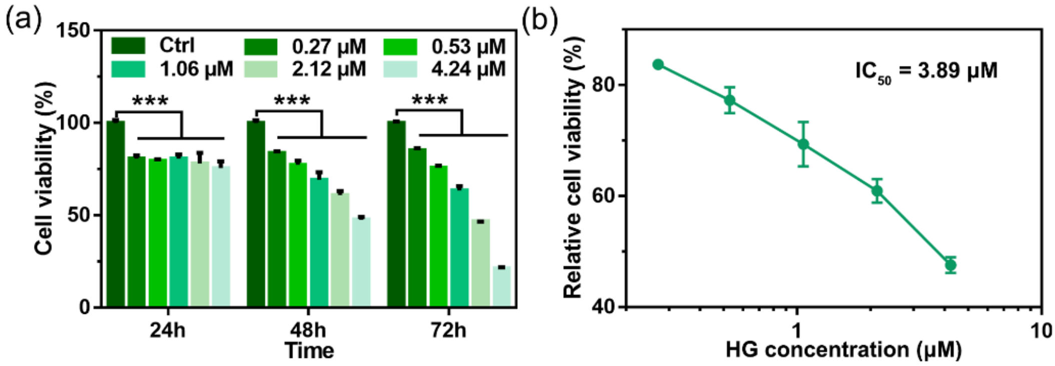

Figure S17. (a) Cell viability of SKOv3-SIK2 cells incubated with **HG** (0-4.24 μM) for 24, 48, or 72 h. (b) Cell viability values at different **HG** concentrations at 48 h in a. Data were expressed as mean ± SD, n = 3. Statistical significance was assessed using one-way ANOVA with Tukey's post-test. $***p < 0.001$.

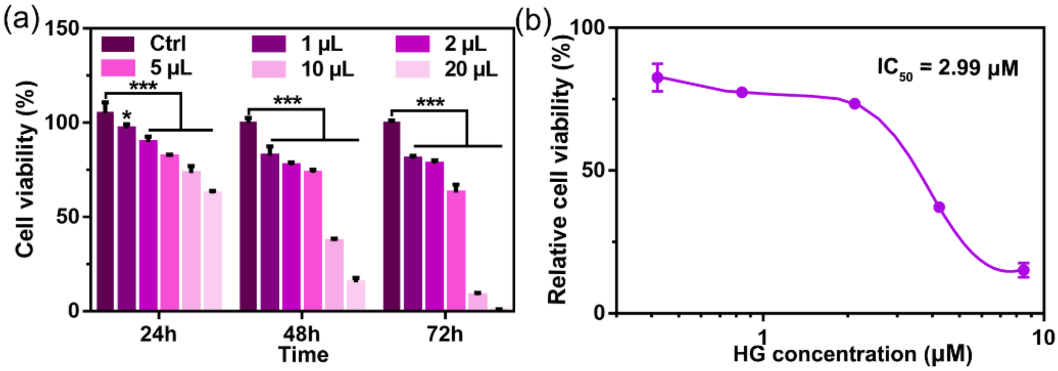

Figure S18. (a) Cell viability of SKOv3-SIK2 cells incubated with different volume of **Gel Nap-S+HG** in 100 μL DMEM culture medium. The original hydrogel **Gel Nap-S+HG** at 1.0 wt% contains 5.3 mM **Nap-S** and 42.4 μM **HG** for 24, 48, or 72 h. (b) Cell viability values at different **HG** concentrations at 48 h in a. Data were expressed as mean ± SD, n = 3. Statistical significance was assessed using one-way ANOVA with Tukey's post-test. $***p < 0.001$.

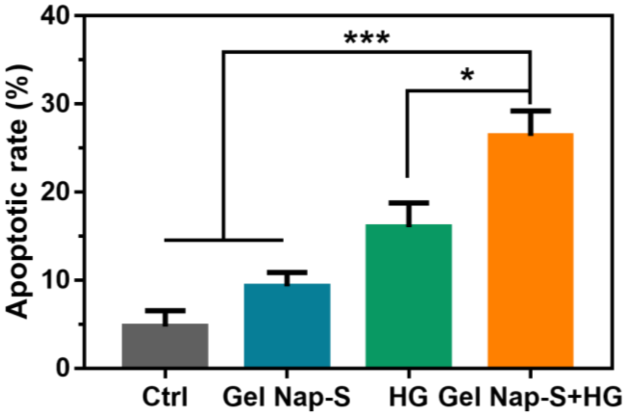

Figure S19. Apoptotic rate after treatment with **Ctrl**, **Gel Nap-S**, **HG**, or **Gel Nap-S+HG** in Fig. 3c. Data were expressed as mean ± SD, n = 3. Statistical significance was assessed using one-way ANOVA with Tukey's post-test. *$p < 0.05$, ***$p < 0.001$.

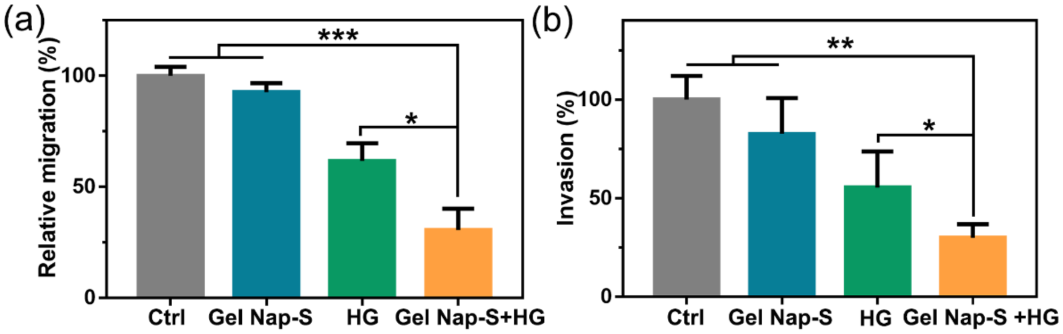

Figure S20. Corresponding statistics of relative (a) migration ratios (b) invasion ratios of SKOv3-SIK2 cells after different treatments in Fig. 3d,e. Data were expressed as mean ± SD, n = 3. Statistical significance was assessed using one-way ANOVA with Tukey's post-test. *$p < 0.05$; **$p < 0.01$; ***$p < 0.001$.

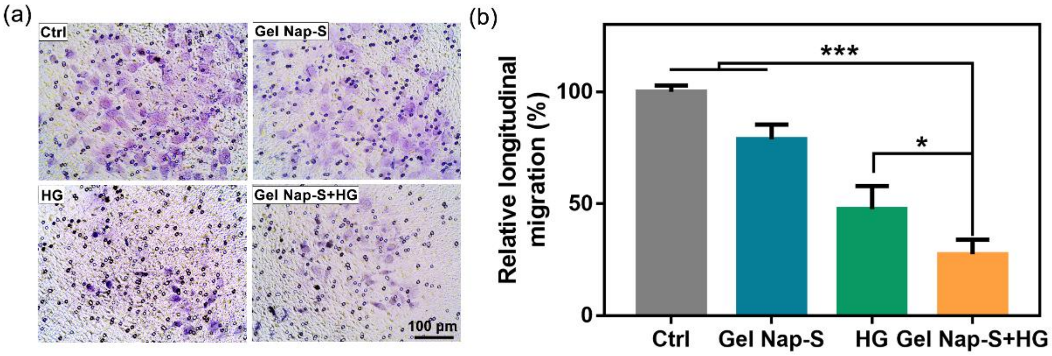

Figure S21. (a) Transwell migration assays of SKOv3-SIK2 cells under different treatments. (b) Corresponding statistics of relative longitudinal migration ratios of SKOv3-SIK2 cells after different treatments in a. Data were expressed as mean ± SD, n = 3. Statistical significance was assessed using one-way ANOVA with Tukey's post-test. *$p < 0.05$, **$p < 0.01$, ***$p < 0.001$.

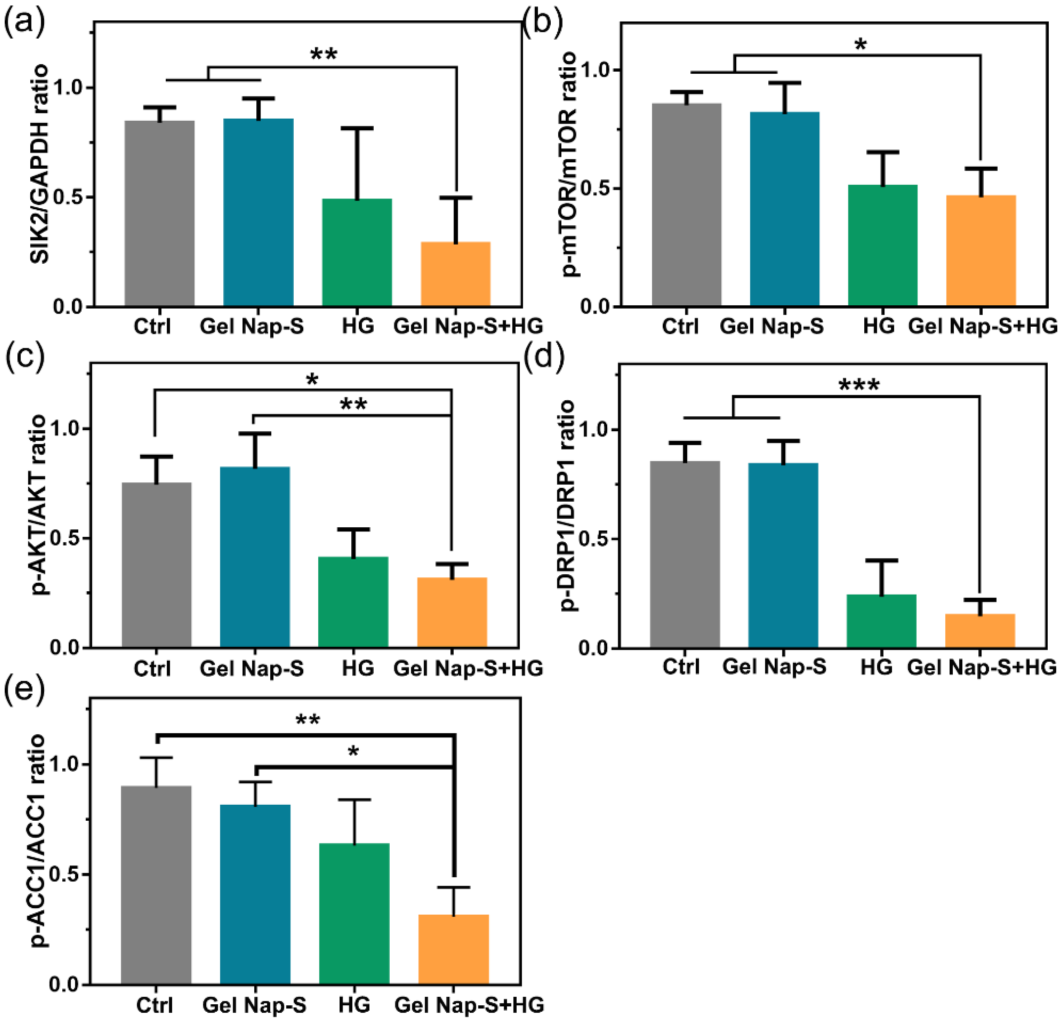

Figure S22. (a-e) Quantification of protein expression levels of SIK2, p-mTOR, p-AKT, p-DRP1, and p-ACC1 in SKOv3-SIK2 cells after different treatments in Fig. 3f. Data were expressed as mean ± SD, n = 3. Statistical significance was assessed using one-way ANOVA with Tukey's post-test. *$p < 0.05$, **$p < 0.01$, ***$p < 0.001$.

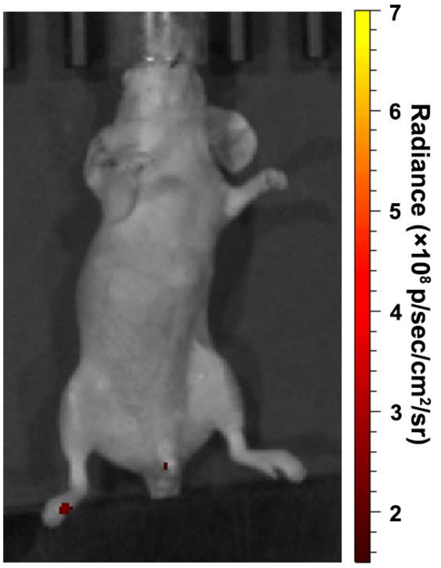

Figure S23. Whole-body fluorescence imaging of living Balb/c nude mouse i.p. injected with SKOv3-SIK2 cells for 20 days.

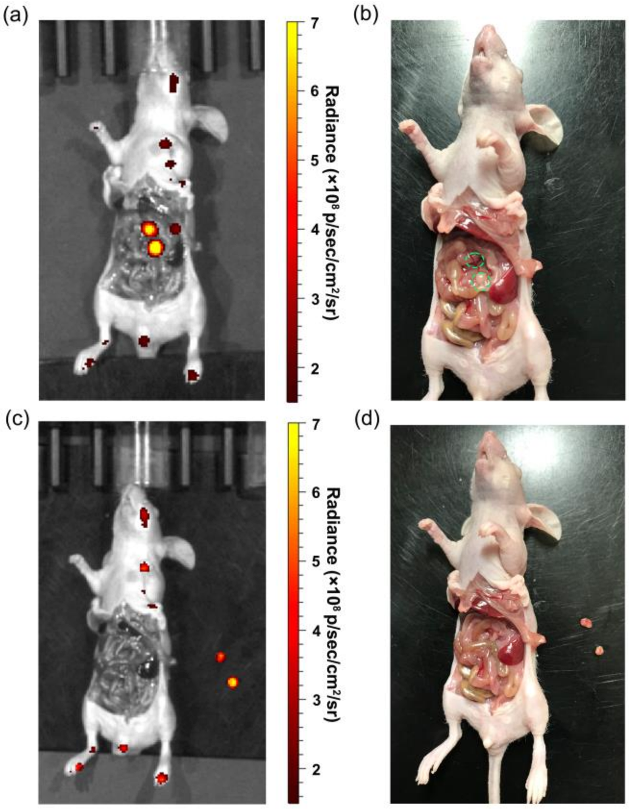

Figure S24. (a) Fluorescence and (b) optical imaging of the peritoneal metastasis mouse in Figure S23 after dissection. (c) Fluorescence and (d) optical imaging of the aforementioned mouse after tumor resection.

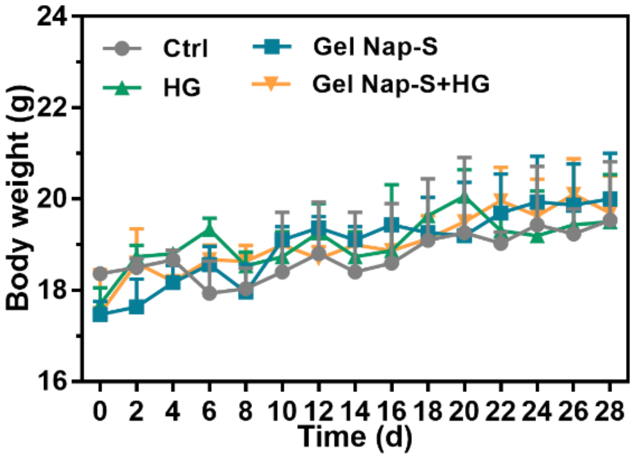

Figure S25. Body weight of SKOv3-SIK2 peritoneal metastasis mice after different treatments during the observation times. Data were expressed as mean ± SD, n = 6. Statistical significance was assessed using one-way ANOVA with Tukey’s post-test.

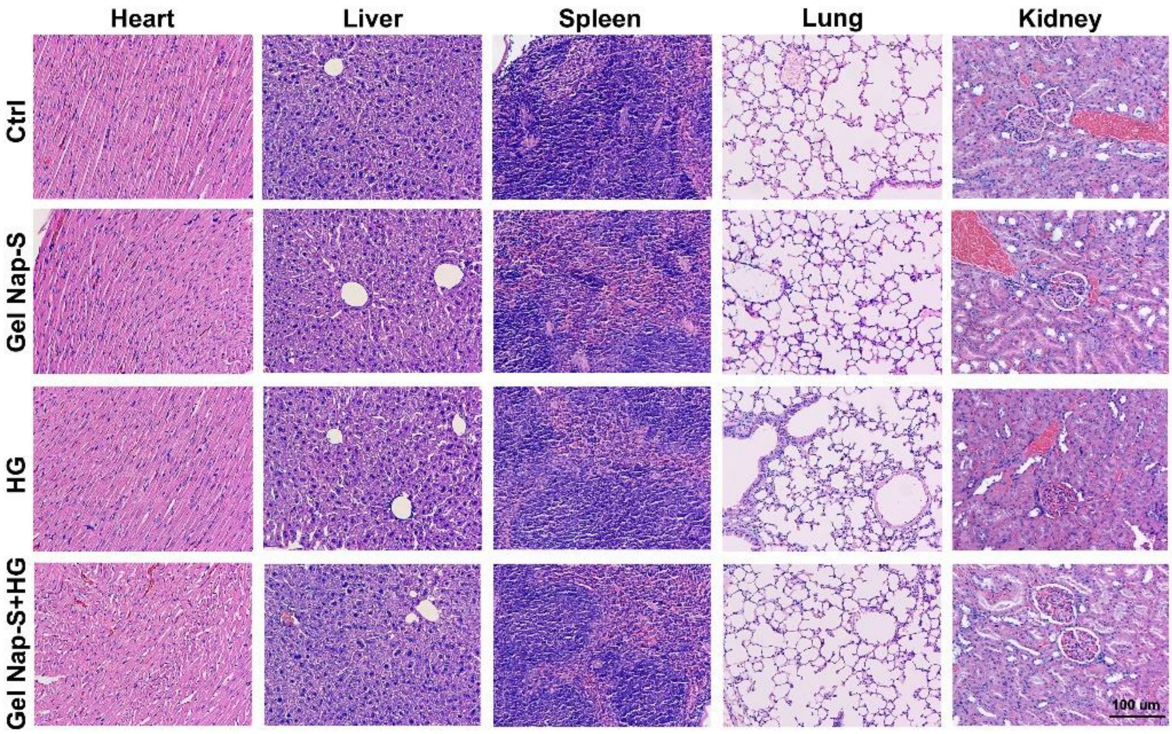

Figure S26. Representative H&E staining of major organs from mice sacrificed at day 8 post different treatment (PBS, **Gel Nap-S**, **HG**, or **Gel Nap-S+HG**).

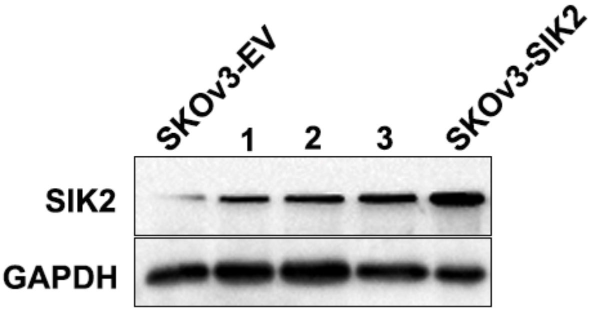

Figure S27. Western blot result of SIK2 expressions in SKOv3-EV cells, ovarian cancer cells from human patient tumor tissues (sample 1, 2, 3), and SKOv3-SIK2 cells.

Table S1. The drug loading content of co-assembled hydrogels with different **HG**/**Nap-S** molar ratios.

**HG**/**Nap-S** (molar ratio)	Drug loading content (wt %)
1 : 500	0.06
1 : 250	0.12
1 : 125	0.24
1: 62.5	0.48
1 : 31.3	0.95
1 : 15.6	1.89
1 : 7.8	3.71
